# Plasmid Viability Depends on the Ecological Setting of Hosts within a Multiplasmid Community

**DOI:** 10.1128/spectrum.00133-22

**Published:** 2022-04-13

**Authors:** Cindy Given, Reetta Penttinen, Matti Jalasvuori

**Affiliations:** a Department of Biological and Environmental Science, Nanoscience Center, University of Jyväskylä, Jyväskylä, Finland; b Department of Biology, University of Turku, Turku, Finland; Griffith University

**Keywords:** plasmid ecology, antibiotic resistance, multiresistance, plasmid evolution, plasmid stability, plasmid-mediated resistance

## Abstract

Plasmids are extrachromosomal genetic elements, some of which disperse horizontally between different strains and species of bacteria. They are a major factor in the dissemination of virulence factors and antibiotic resistance. Understanding the ecology of plasmids has a notable anthropocentric value, and therefore, the interactions between bacterial hosts and individual plasmids have been studied in detail. However, bacterial systems often carry multiple genetically distinct plasmids, but dynamics within these multiplasmid communities have remained unstudied. Here, we set to investigate the survival of 11 mobilizable or conjugative plasmids under five different conditions where the hosts had a differing ecological status in comparison to other bacteria in the system. The key incentive was to determine whether plasmid dynamics are reproducible and whether there are tradeoffs in plasmid fitness that stem from the ecological situation of their initial hosts. Growth rates and maximum population densities increased in all communities and treatments over the 42-day evolution experiment, although plasmid contents at the end varied notably. Large multiresistance-conferring plasmids were unfit when the community also contained smaller plasmids with fewer resistance genes. This suggests that restraining the use of a few antibiotics can make bacterial communities sensitive to others. In general, the presence or absence of antibiotic selection and plasmid-free hosts (of various fitnesses) has a notable influence on which plasmids survive. These tradeoffs in different settings can help explain, for example, why some resistance plasmids have an advantage during a rapid proliferation of antibiotic-sensitive pathogens whereas others dominate in alternative situations.

**IMPORTANCE** Conjugative and mobilizable plasmids are ubiquitous in bacterial systems. Several different plasmids can compete within a single bacterial community. We here show that the ecological setting of the host bacteria has a notable effect on the survival of individual plasmids. Selection for opportunistic genes such as antibiotic resistance genes and the presence of plasmid-free hosts can determine which plasmids survive in the system. Host bacteria appear to adapt specifically to a situation where there are multiple plasmids present instead of alleviating the plasmid-associated fitness costs of individual plasmids. Plasmids providing antibiotic resistance survived under all conditions even if there was a constant migration of higher-fitness plasmid-free hosts and no selection via antibiotics. This study is one of the first to observe the behavior of multiple genetically different plasmids as a part of a single system.

## INTRODUCTION

Bacterial cells serve as vehicles for various types of genetic replicators. In addition to chromosomes, these include transposing elements, plasmids, conjugative elements, and viruses. Many of these replicators contain genes that can entirely dictate whether the hosting cellular vehicle (host) prevails in its present environment (1–3). Plasmids are common genetic molecules of bacteria that (usually) replicate separately from the host chromosome (4). Plasmids are of notable anthropocentric importance due to their clinically relevant features, mainly antibiotic resistance and virulence factors (5, 6). The presence of a (conjugative) plasmid in a particular strain can therefore translate into both the development of a disease and treatment failure. Conjugative plasmids encode a machinery that facilitates the transfer of the element into surrounding bacterial cells (7). Mobilizable plasmids do not encode conjugation machineries but utilize the machineries encoded by other elements to hitchhike along the way and disperse from one cell to another (8). Among Gram-negative pathogens, conjugative and mobilizable plasmids are the most common genetic replicators mediating multidrug resistance (9–11).

Bacteria carry various types of plasmids that can be divided into incompatibility groups according to their capability to stably coexist in a single cell line (12, 13). The general prevalence of plasmids has been puzzling to explain in the absence of selection for plasmid-carried genes, since in new hosts, plasmids often induce fitness costs (14). These costs, however, may be rapidly alleviated by adaptive mutations (14–16). Conjugation rates also differ, and hence, the plasmid-associated costs to host fitness may be balanced by their ability to invade surrounding cells (5). Under certain conditions such as active predation by protozoa, plasmids’ survival appears to be dependent on their ability to conjugate (2). Adaptation to specific plasmids can also cause bacteria to become more permissive to other plasmids and the evolved plasmids, consequently, to be a lesser burden in alternative hosts (16, 17). Different plasmids that have adapted to hosts of different bacterial species can come in contact later on and form successful novel plasmid combinations (18, 19) and hence form new multiresistant strains. Altogether, these studies draw a picture in which several factors contribute to the means by which plasmids and their hosts can form a stable long-term companionship. These factors together may resolve the so-called plasmid paradox—in other words, explain why plasmids do not disappear when we withdraw the selection for specific plasmid-carried genes (as discussed in reference 14).

In reality, however, bacterial cells are often in an environment where multiple plasmids are continuously present and new plasmids migrate within new hosts. Extended-spectrum-beta-lactamase (ESBL)-conferring plasmids are substantially diverse in their specific genetic characteristics as well as in their incompatibility groups and mobility types (9, 20). Therefore, bacteria face situations where a variety of plasmids compete within host cells that are situated in the same community (21). Many basic questions in this domain remain mostly unstudied. Are there, for example, characteristics that make certain plasmids outcompete others in a multiplasmid community, and if so, how stochastic are these dynamics? Are there specific adaptations in the host in a situation where multiple genetically different plasmids occupy the community in comparison to often-studied situations where adaptations can focus on alleviating costs of singular plasmids (21)? Is there always a single winner in a certain set of plasmids, or can the winner be dependent on the ecological setting of the host? Indeed, either new plasmid-free bacterial hosts may form within the community as some of them lose plasmids or they may migrate in from other environments. These new migrants may also have higher fitness than members of the original community, and hence dispersal to new hosts can become essential for plasmid survival. To our knowledge, no studies have investigated the dynamics within such multiplasmid communities with more than three different plasmids and rarely under alternating ecological conditions. Exploring the answers to these questions served as an incentive for this study.

Here, we set out to investigate a set of plasmids with various characteristics in differing ecological settings. A total of 11 different plasmids originating from clinical bacterial isolates were transferred to an isogenic background (Escherichia coli) in their natural combinations. This generated six bacterial strains with various plasmid contents that were subjected together to a 42-day-long serial coculture experiment in five different ecological settings. The results show that some plasmids prevail under specific conditions while they almost completely disappear under others. The host adaptation appears to have resulted from general adaptation to culturing conditions, or perhaps to the presence of multiple plasmids, instead of alleviating plasmid-specific costs. Overall, there appear to be tradeoffs that may play a role in the success of some plasmids, e.g., in the presence of hosts with higher fitness.

## RESULTS

We set up a serial culture microcosm experiment where each system was seeded with 11 plasmids that originated from ESBL-encoding Escherichia coli strains in addition to a well-characterized conjugative plasmid, RP4. The plasmids were transferred in their natural combinations to laboratory E. coli K-12 strain HMS174 in order to even out the competitive advantages in the original hosts (22). Some of the studied plasmids are conjugative, and some are mobilizable (indicating that they are transferred along with a conjugative plasmid). In all plasmid combinations, at least one of the plasmids encodes a beta-lactamase that provides resistance to beta-lactam antibiotics. Plasmid RP4 was included as it has been utilized in various studies where RP4-encoded conjugation machinery has been employed to deliver CRISPR systems to antibiotic-resistant bacteria (23, 24). As such, RP4’s capability to compete among ESBL plasmids provides estimates for its potential to modify bacterial communities with any introduced CRISPR tools. However, RP4 contained a kanamycin resistance gene, which, due to the experimental design, had to be inactivated prior to the microcosm experiments. Further, the ability of the plasmids to coexist in a single cell was not determined, and hence, the observed dynamics are determined solely on a community level. In a simple drop test, the strain containing plasmid pEC16pl2 inhibited the growth of other strains. However, in a 5-day-long preliminary experiment, other plasmids persisted with pEC16pl2, and therefore, it was included in the setup. Plasmids and their key features are listed in Table 1.

**TABLE 1:** Plasmid-harboring strains used in the experiment^*a*^

Strain	Plasmid(s)	Plasmid size (bp)	Inc type(s)	MPF type	MOB class	Beta-lactamase identified	Other resistance genes	Reference(s)
E. coli HMS174 (plasmid free), Rif^r^								
E. coli HMS174(pEC3), Rif^r^ Amp^r^	pEC3pl1	91,885	IncB/O/K/Z	MPFI	MOBP	*bla*_TEM-1C_	*strA*, *strB*, *sul2*	22
	pEC3pl2	59,192 (59,192)^*b*^	IncI2	MPFT	MOBP			22
E. coli HMS174(pEC13), Rif^r^ Amp^r^	pEC13	71,656	IncFII	MPFF	MOBF	*bla*_CTX-M-14_		22
E. coli HMS174(pEC14), Rif^r^ Amp^r^	pEC14pl1	143,590	IncFII, IncQ1, IncP, IncFIB	MPFF	MOBF	*bla*_TEM-1B_	*strA*, *strB*, *aadA1*, *mph(B)*, *sul1*, *sul2*, *tet(A)*, *dfrA1*	22
	pEC14pl2	87,848 (87,666)^*b*^	IncI1	MPFI	MOBP			22
	pEC14pl3	80,057	IncFII	MPFF	MOBF			22
E. coli HMS174(pEC15), Rif^r^ Amp^r^	pEC15pl1	87,811 (87,767)^*b*^	IncI1	MPFI	MOBP			22
	pEC15pl2	38,611	IncX1	MPFT	MOBQ	*bla*_TEM-52B_		22
E. coli HMS174(pEC16), Rif^r^ Amp^r^	pEC16pl1	94,325 (95,380)^*b*^	IncI1	MPFF	MOBP	*bla*_SHV-12_		22
	pEC16pl2^*c*^	7,939	ColRNAI		MOBP			22
E. coli HMS174(RP4-s.g.2), Rif^r^ Amp^r^ Tet^r^ ΔKan	RP4	60,096	IncP-1α	MPFT	MOBP	*bla*_TEM-1_		8, 40
E. coli HMS174(pET24), Rif^r^ Kan^r^	pET24^*d*^	5,236						

a MPF, mating pair formation; MOB, mobility.

b Alterations to plasmid size due to shufflon area are indicated in parentheses.

c Nonconjugative mobilizable plasmid.

d Nonconjugative plasmid.

**FIG 1:**
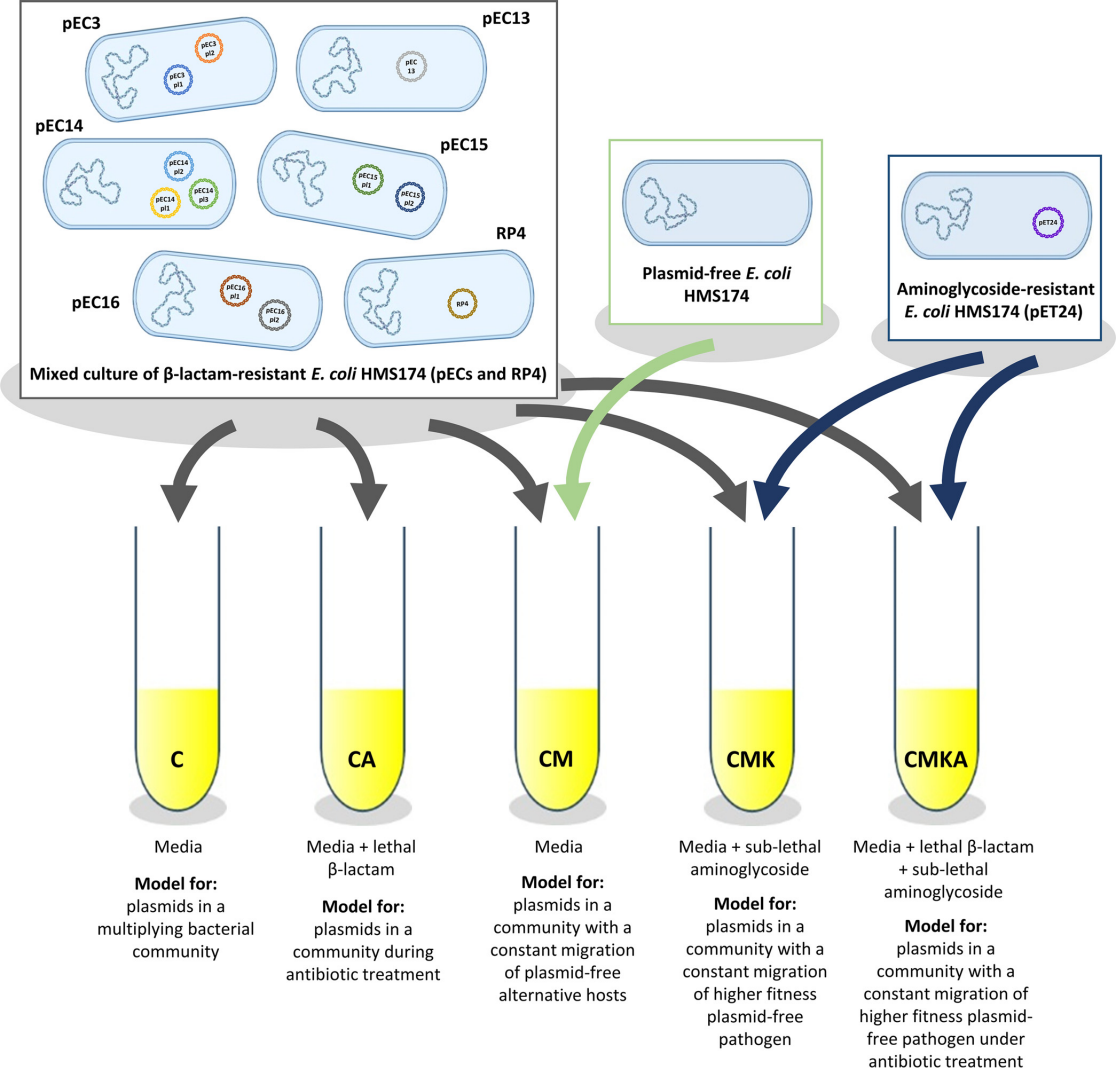
The experimental design of the serial culture experiment. Ampicillin was used for beta-lactam and kanamycin for aminoglycoside. The cultures were refreshed 41 times (*n* = 4/treatment).

The plasmid-harboring bacterial strains were mixed together for the microcosms that modeled five different ecological conditions (Fig. 1). Each microcosm was refreshed 41 times, resulting in roughly 320 bacterial generations. Each experimental condition was carried out in four independent replicates. In the first setup, bacteria were cultivated and refreshed as a community (designated C for community). In the second setup, the community (CA, community + ampicillin) was subjected to continuous beta-lactam selection (thus representing an environment during antibiotic treatment). In the third setup (CM, community + migration), the bacterial community was continuously supplemented with plasmid-free migrating hosts in a 1:10 ratio with the community. These migrants can be invaded by the plasmids. In the fourth setup (CMK, community + migration + kanamycin), the community was continuously supplemented with migrating host bacteria that have higher fitness than the original plasmid hosts in an attempt to model the presence of rapidly proliferating plasmid-free pathogens. In the fifth setup (CMKA, community + migration + kanamycin + ampicillin), the community was treated with an antibiotic in the presence of a migrating antibiotic-susceptible pathogen which could restore its full replication rate by receiving a resistance plasmid from the resistant community.

**FIG 2:**
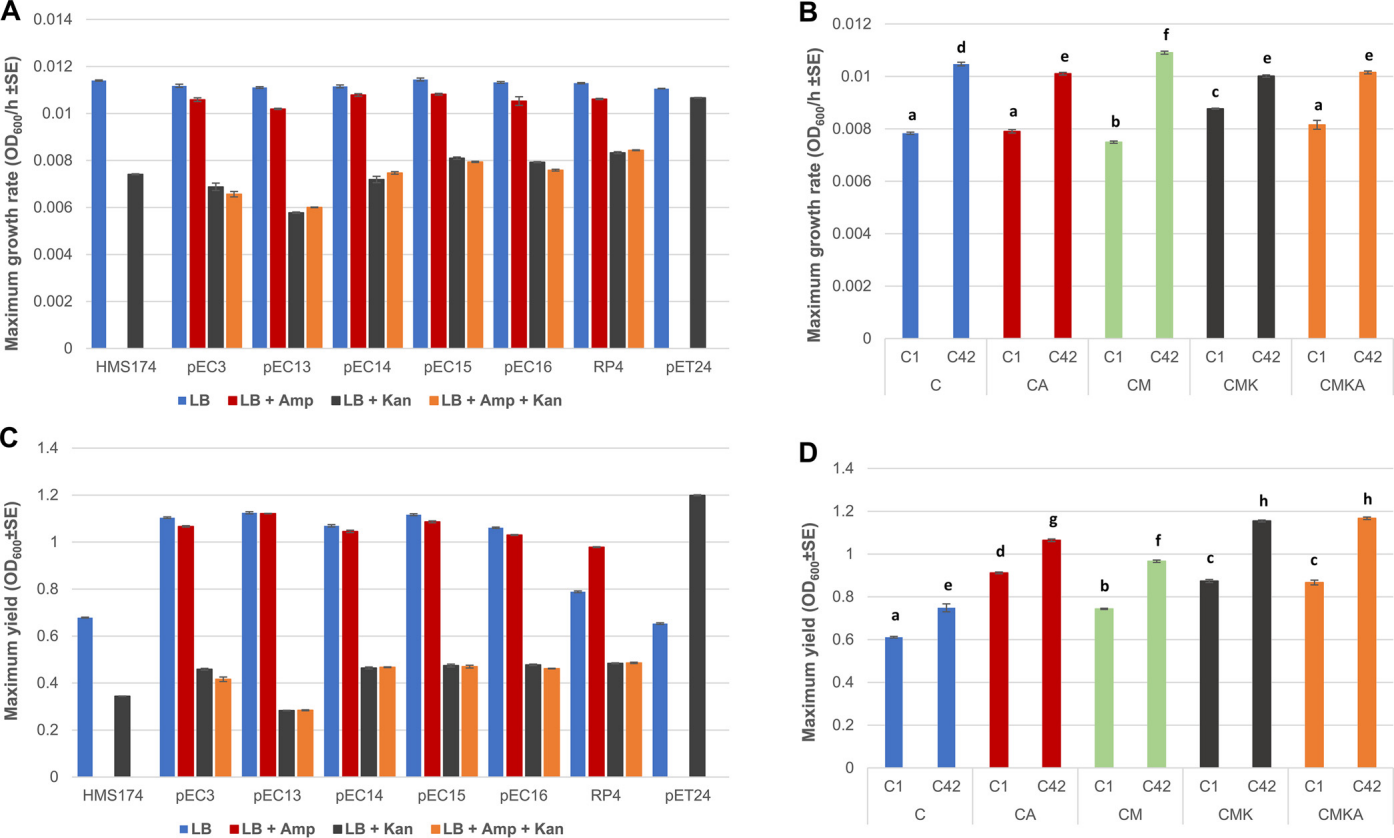
Maximum growth rates and maximal optical density (600 nm) of cultures of each individual strain (A and C) and communities under different treatments at the start and end of the experiment (B and D). Maximum growth rate indicates a maximum change in optical density in 1 h during the 24-h measurement. C1 and C42 refer to the serial culture experiment after day 1 and day 42, respectively. Statistical difference of growth rates and optical density was determined using two-way ANOVA (*P* < 0.05). The growth rate for the CMK communities at the starting point was the highest (*P* < 0.01, 2-way ANOVA; *post hoc* test, Tukey HSD), whereas no difference in the growth rate was shown between C, CA, and CMKA (*P* = 0.067, 2-way ANOVA; *post hoc* test, Tukey HSD). At the endpoint, significant difference was observed among all treatments (*P* < 0.01, 2-way ANOVA; *post hoc* test, Tukey HSD) (B). Optical densities of the communities at the endpoint were higher than at the starting point between all treatments (*P* < 0.01, 2-way ANOVA; *post hoc* test, Tukey HSD) (D). Different letters indicate statistically significant differences in results.

We measured the growth rates and maximum population densities for individual plasmid-hosting strains prior to starting the experiment as well as for the evolved communities (Fig. 2). All plasmids had nonsignificant effects on the replication rate of the hosts (Fig. 2A). During the course of the experiment, the maximum growth rate (i.e., the maximal rate at which optical density increased over 1-h time windows during the 24-h measurement) of the community increased significantly under all conditions (Fig. 2B). Interestingly, the presence of plasmids increased the optical density of the population (Fig. 2C). This may derive either from the increased biofilm formation due to plasmids or from increased population density. Evolved communities also showed increases in optical density compared to the starting point (Fig. 2D). The kanamycin concentration utilized in the experiment was selected to make the migrating bacterium [HMS174(pET24)] have both a higher replication rate and higher density than the kanamycin-sensitive strains. This was done to model the presence of a higher-fitness plasmid-free host. We also measured the conjugation rates for all plasmid-harboring hosts by determining the frequency at which each bacterium was able to transfer their beta-lactamase resistance to a susceptible recipient (Table 2). While this does not reveal the transfer rates for nonresistance plasmids, previous study with these plasmids indicated that all plasmids were cotransferred together (22). However, in this experimental setup, it is likely that transfer rates of individual plasmids vary depending on the plasmid content of any particular cell.

**TABLE 2:** Average conjugation frequency per donor cell after 2 h

Plasmid group	Mean conjugation frequency per donor cell
pEC3	3.70E−06
pEC13	8.06E−05
pEC14	7.22E−07
pEC15	2.87E−06
pEC16	3.29E−05
RP4	1.50E−04

**FIG 3:**
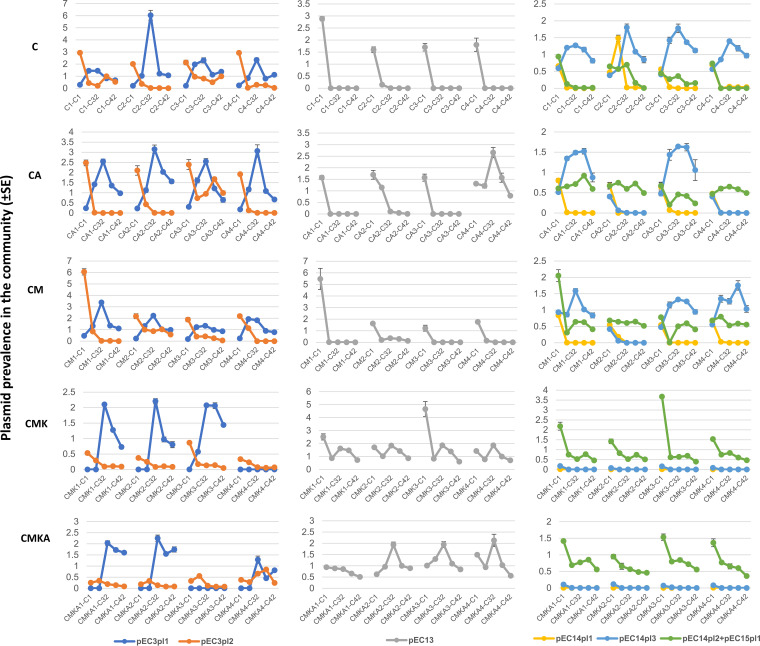
Prevalence of plasmids pEC3pl1, pEC3pl2, pEC13, pEC14pl1, pEC14pl3, and pEC14pl2+pEC15pl1 under different conditions as normalized with the gene for 16S rRNA. Plasmid prevalence was followed over 42 cycles of the serial culture experiment. Culture conditions are depicted in Fig. 1. Alternative visualizations of the results are available in Data Set S1, sheet D.

**FIG 4:**
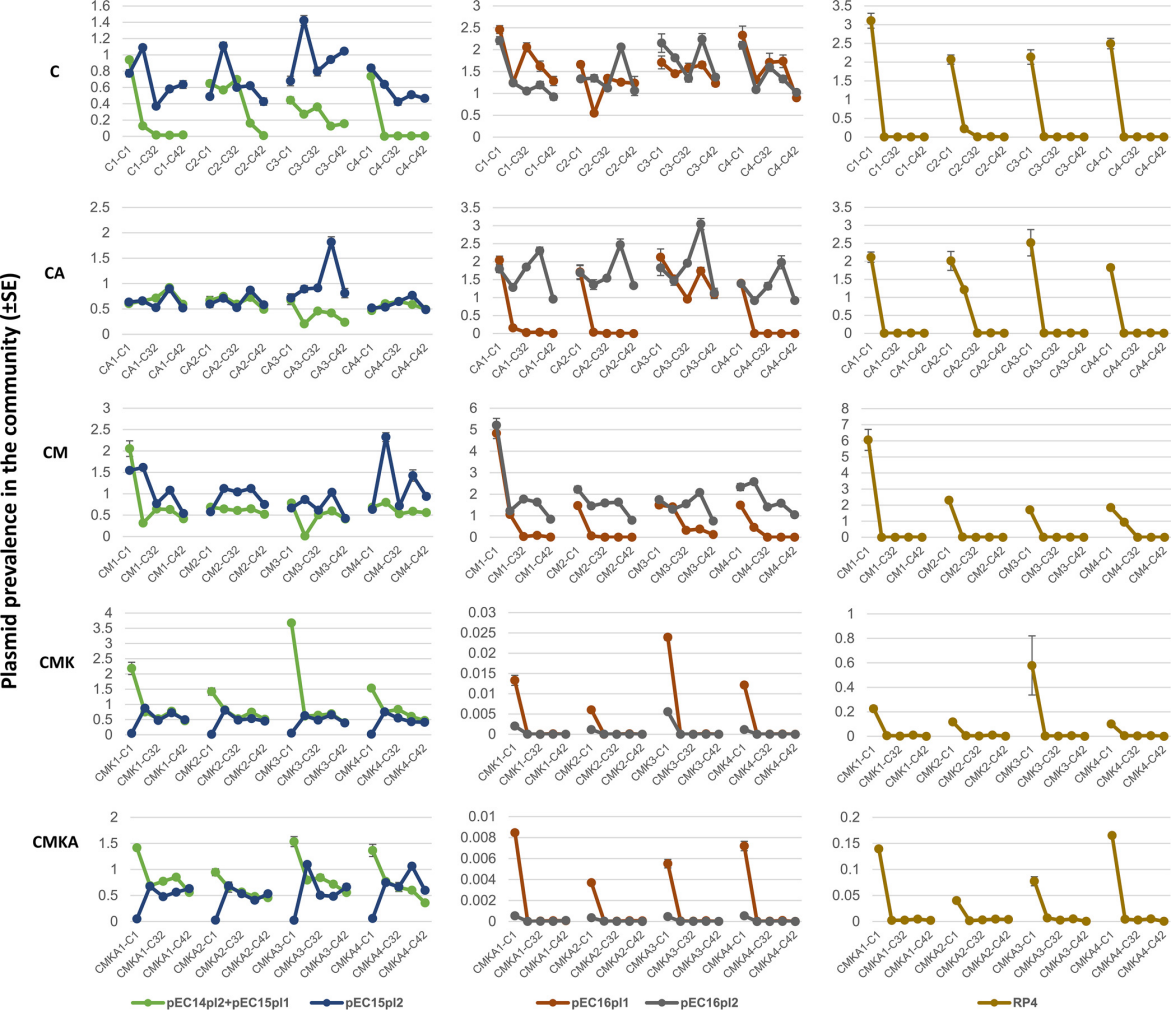
Prevalence of plasmids pEC14pl2+pEC15pl1, pEC15pl2, pEC16pl1, pEC16pl2, and RP4 under different conditions as normalized with the gene for 16S rRNA. Plasmid prevalence was followed over 42 cycles of the serial culture experiment. Culture conditions are depicted in Fig. 1. Alternative visualizations of the results are available in Data Set S1, sheet D.

Tracking the abundance of multiple plasmids with no distinct selective genes can be challenging. As such, we employed quantitative PCR (qPCR) with specific primers against each plasmid (excluding pEC14pl2 and pEC15pl1, which were almost identical and hence were amplified with a single primer pair). Primer sequences and primer analysis for pure cultures grown under the same conditions as in the experiment are presented in Data Set S1, sheets E and F, in the supplemental material. Total DNA was isolated at five time points (1, 14, 32, 35, and 42 days) during the serial culture experiment, and the relative quantity of each plasmid was determined against a 16S rRNA genetic marker. qPCR measurements for each plasmid were conducted in three replicates. Figures 3 and 4 represent the evolution of plasmid prevalence over time (see Data Set S1, sheet D, for original data). The majority of the 11 plasmids behaved similarly in all independent replicates within a particular experimental setup. However, pEC13 almost completely disappeared in all but one CA replicate, where it was well represented, and pEC14pl3 almost completely disappeared in two CA replicates and in one CM replicate. Notably, pEC13 also appeared to be less fit in the presence of kanamycin than other plasmids. Yet, it survived best under conditions containing sublethal kanamycin concentrations. This nevertheless suggests that plasmid dynamics are generally consistent in a certain ecological setting.

We took a closer look at general features of plasmids under each of the ecological conditions to reveal characteristics that may be advantageous for plasmid survival (Table 3). Mobilizable and conjugative plasmids can be grouped with a MOB classification system that bases its groups on relaxase genes (25). Relaxase itself is responsible for both initiating and terminating conjugative transfer of a plasmid. Conjugative plasmids can also be classified by the protein complex that mediates the mating pair formation (MPF) with a recipient cell (26).

**TABLE 3:** Persistence of different mobility and mating pair formation classes

Treatment	Mobility		*P* value	Mating pair formation			*P* value
	MOBP	MOBF		MPFI	MPFT	MPFF	
C	Most plasmids persisted	All but one plasmid disappeared	0.001	Most plasmids persisted	Most plasmids persisted	Most plasmids persisted	0.919
CA	Most plasmids persisted	Most plasmids decreased and disappeared	0.001	All plasmids persisted	Most plasmids disappeared	Half of the plasmids disappeared	≤0.01
CM	Most plasmids persisted	All but one plasmid disappeared	0.005	All plasmids persisted	Most plasmids decreased and disappeared	Most plasmids decreased and disappeared	≤0.01
CMK	Most plasmids persisted	One out of three plasmids persisted	0.226	All plasmids persisted	All plasmids persisted	Most of the plasmids disappeared	≤0.01
CMKA	Most plasmids persisted	Most plasmids persisted	0.142	All plasmids persisted	All plasmids persisted	Most plasmids disappeared	≤0.01

Mobility type of a plasmid (MOB) did not affect the persistence of different plasmids in any of the treatments (C, *P* = 0.114; CA, *P* = 0.063; CM, *P* = 0.189; CMK, *P* = 0.319; CMKA, *P* = 0.355; one-way analysis of variance [ANOVA]; *post hoc* test, Tukey honestly significant difference [HSD]). On the other hand, the significant difference between types of mating pair formation (MPF) was shown in CM, CMK, and CMKA treatments but not in C and CA (C, *P* = 0.586; CA, *P* = 0.315; one-way ANOVA; *post hoc* test, Tukey HSD). At the endpoint of the experiment with CM, all plasmids with MPFI persisted in the system while plasmids with MPFT and MPFF generally decreased and/or disappeared from the system (*P* = 0.005, one-way ANOVA; *post hoc* test, Tukey HSD). In CMK, all plasmids with MPFI and MPFT persisted in the system while most of the plasmids (except pEC13) with MPFF disappeared. However, statistically, MPFI showed significant difference in plasmid prevalence compared to MPFT and MPFF (*P* = 0.007, one-way ANOVA; *post hoc* test, Tukey HSD), but there was no difference between MPFF and MPFT (*P* = 0.997). A similar result is observed in CMKA, i.e., all plasmids with MPFI and MPFT persisted in the system while most of the plasmids (except pEC13) with MPFF disappeared from the system (*P* ≤ 0.01, one-way ANOVA; *post hoc* test, Tukey HSD), and no significant difference was observed between MPFF and MPFT (*P* = 0.894). It must be noted that, while there are potentially relevant benefits in different MOB and MPF systems under different conditions, the plasmids vary in multiple characteristics, and hence, these results should be approached very cautiously. For incompatibility types, IncB can persist in the system better than other Inc types and IncP did not survive under any condition (see Data Set S1, sheet J). Further, it is known that a beta-lactamase-producing bacterium can support antibiotic-sensitive cheaters in its vicinity (27). The beta-lactam resistance gene was not necessary for plasmid survival in the presence of ampicillin (CA and CMKA) as long as some bacteria in the community retained resistance. However, some beta-lactamase-encoding plasmids remained at comparably high levels also in communities where beta-lactam antibiotic was absent.

**FIG 5:**
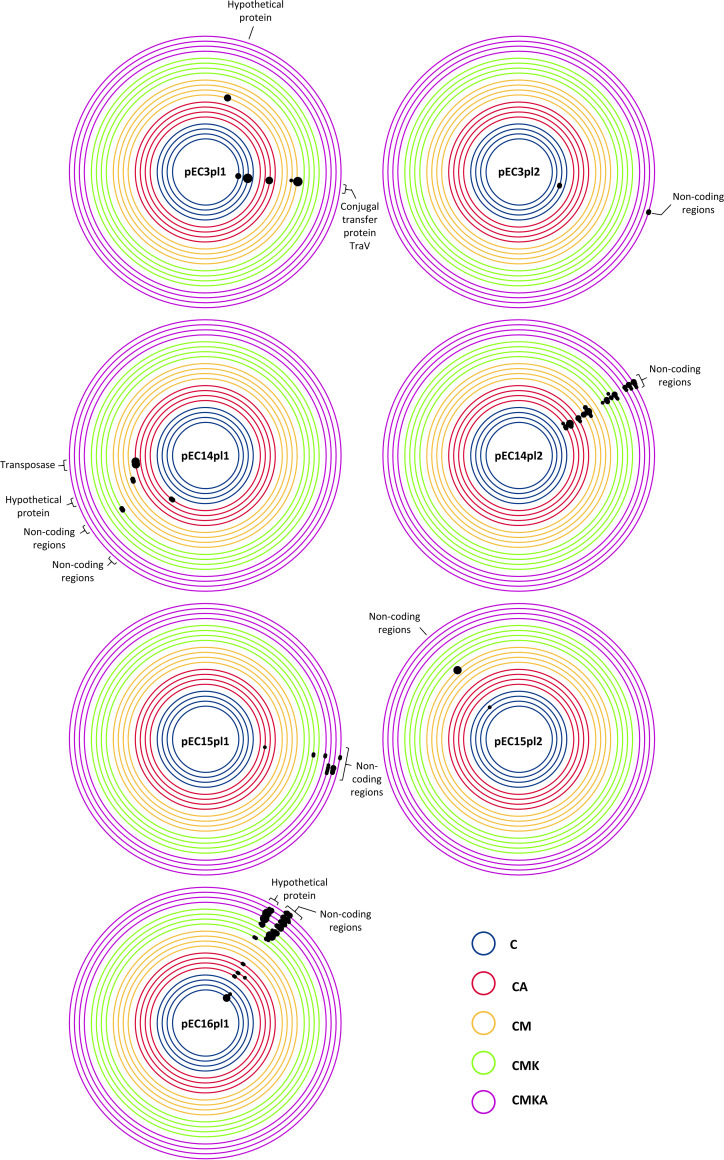
Observed mutations in plasmids after 42 cycles in the serial culture experiment (∼320 generations). Only plasmids with mutations are shown. The black dots mark the mutation region and its frequency; the bigger the black dot, the higher the mutation frequency (for exact values and mutation types, see Data Set S1, sheet G).

**FIG 6:**
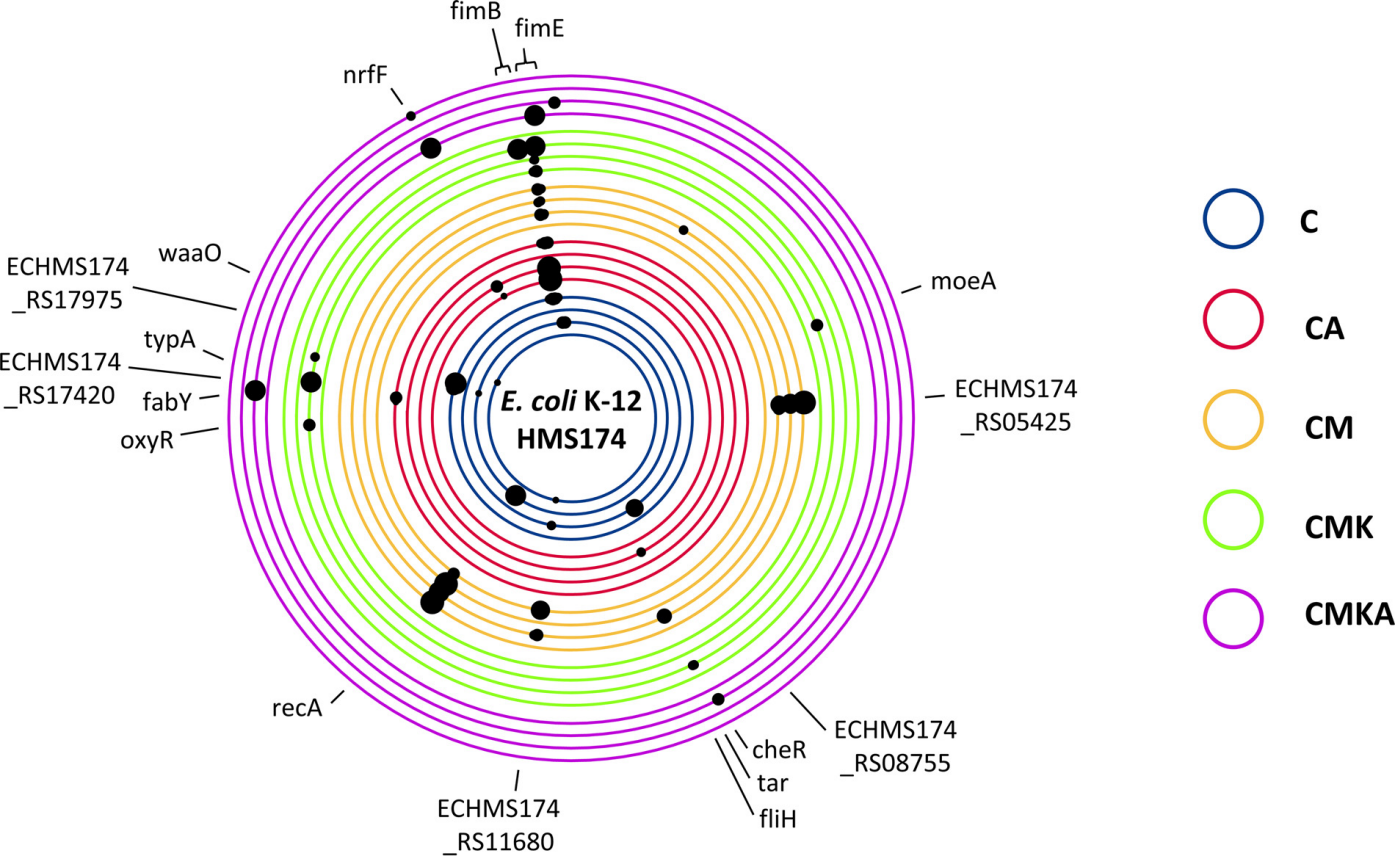
Observed mutations in the host chromosomes obtained from the community after 42 cycles in the serial culture experiment (∼320 generations). Mutations in noncoding regions are not labeled. The black dots mark the mutation region and its frequency; the bigger the black dot, the higher the mutation frequency (for exact values and mutation types, see Data Set S1, sheet G).

We also investigated potential evolutionary changes in the plasmids. Sequencing of the endpoint communities revealed some mutations that repeatedly appeared during the serial culture experiment. Most of the mutations in plasmids were located in noncoding regions or genes with unknown functions, excluding pEC3pl1, in which mutations within the gene for conjugal transfer protein V (TraV) appeared in multiple communities (Fig. 5). Genetic changes in noncoding regions were close to transposases or shufflon regions that are known to be less stable due to continuous DNA “shuffling” (28). The bacterial host consistently accumulated mutations in genes for type 1 fimbria regulatory protein (FimE) and under certain conditions (CM) a single nucleotide variant in the gene for RecA (Fig. 6). The low number of mutations in the CMKA community is likely to derive from the continuous supplementation with nonevolved HMS174 that had higher fitness than the plasmid-harboring strains. This produced constant migration of naive mutation-free hosts that possessed the advantageous nontransmissible plasmid pET24. No mutations in four conjugative plasmids (pEC13, pEC14pl3, pEC16pl2, and RP4) and pET24 were detected in any of replicate communities. Further, it must be noted that some reads could be mapped at the endpoint for all plasmids and that the number of reads matches the qPCR data (Data Set S1, sheet H). This suggests that the apparently disappeared plasmids partly or wholly were still existing in the community but only at very low levels compared to the starting point. Additionally, it is possible that some plasmids have recombined with other plasmids or with the chromosome over the course of the experiment.

## DISCUSSION

Plasmids are ubiquitous genetic elements of bacteria. They are responsible for several notable phenotypes of their hosts, such as antibiotic resistance or increased virulence. Arguably, these opportunistic genes that provide immense benefits under only specific conditions are likely to accumulate in horizontally transferring elements. This is because genes in mobile elements are most likely to find their way to the high-fitness hosts of the community (1, 18, 29). Nonetheless, not all plasmids have clearly opportunistic genes (30), and plasmids coexist in different ecological settings where only certain plasmid backbones may find success. The potential effects that these conditions have on the survivability or dominance of plasmids in the presence of multiple competitors, to our knowledge, have not been studied directly. We set out to investigate how plasmids thrive in the presence and absence of selection for opportunistic genes (antibiotic resistance) when there is or is not a migrating plasmid-free host with higher or equal fitness available. The studied plasmids represent various incompatibility types, mobility groups, and conjugation systems and hence resemble a situation where different plasmids exist in a shared environment.

Certain plasmids were disappearing regardless of the tested setup. Plasmid pEC14pl1 was almost completely lost in all experiments despite containing a resistance gene against ampicillin. It is by far the largest plasmid (143 kb) in this experiment. While the plasmid cost appears to have only a minor effect on the growth rate or host density in the media used in the experiment, it is still possible that even subtle costs that derive from its overall size and nine resistance genes accumulate in a prolonged experiment. This supports the line of reasoning where those plasmids that have accumulated large numbers of opportunistic genes are more dependent on specific selection on plasmid-carried genes than smaller plasmids (11). However, and similarly to pEC14pl1, RP4 also almost completely disappeared from all communities despite being relatively small (60 kb). RP4 is an IncP plasmid that has a wide host range, and it had the highest conjugation rate of all the plasmids of this experimental setup. Yet, it caused a greater reduction in the total population density than other plasmid combinations, and therefore, in a serial culture experiment, it is likely to dilute out. However, the fitness cost of RP4 has been shown to be readily ameliorated in a Pseudomonas sp. host after mutations in genes for accessory helicases (16). Similar helicases exist in E. coli used in the study, but no such mutations were observed at the end of the experiment. This suggests that, while mutations may alleviate costs, if alternative plasmids are present that are initially less costly to the host and provide similar opportunistic genes (antibiotic resistance), they are likely to rapidly outcompete maladapted plasmids and hence probably reduce the chances for ameliorative mutations to establish in the community. IncP plasmids are also noted to be rare among enterobacterial pathogens (9), and hence, the observed inability to survive against more common (narrow-host-range) plasmids (such as IncF and IncI) is to be expected. If the system had alternative hosts besides E. coli, the variety of potential hosts (which are generally unsuitable for the here-used pEC plasmids) may have allowed it to prevail (30, 31). All other plasmids survived under at least some conditions.

The plasmids in this study were seeded into the experiment in their natural combinations. It could be anticipated that these plasmids had already adapted to coexistence in their original hosts. However, in only some setups pEC15 and pEC16 plasmids remained at similar levels throughout the experiment (although it must be noted that our quantification method did not allow determining whether they existed in the same cell). As such, previous coexistence appears to readily dissolve, and potentially new plasmid combinations form.

Interestingly, pEC16pl2 is a small mobilizable plasmid that encodes a (putative) microcin that is lethal to surrounding cells unless it carries the plasmid. HMS174 with pEC16 inhibits the growth of HMS174 harboring any of the other plasmid combinations. Intrinsically, it appears reasonable to expect pEC16pl2 to become dominant in the community. Nevertheless, preliminary experiments demonstrated that pEC16pl2 does not replace other plasmids from the system even in the absence of antibiotics, and as such, we retained it in the experimental setup to evaluate the plasmid’s long-term viability. Indeed, in the absence of the simulated presence of a pathogen (i.e., sublethal kanamycin and kanamycin-resistant E. coli), the number of pEC16pl2 plasmids remained among the highest. However, the introduction of kanamycin and a kanamycin-resistant host (i.e., simulated proliferating pathogen) caused it to rapidly disappear. This indicates a clear tradeoff where change in the ecological setting turns the plasmid’s fitness completely around. The initially coexisting plasmid partner of pEC16pl2 (i.e., pEC16pl1) was stable only in the refreshed community (C), suggesting that pEC16pl2 prevalence in other systems was independent of pEC16pl1.

Plasmids are generally divided into incompatibility groups where two plasmids of the same type cannot coexist in a single cell line. Here, plasmids pEC13 and pEC14pl3 both belong to IncFII and generally prevailed in only those cultures where the other was absent. pEC13 became the dominant IncFII plasmid under conditions where the simulated pathogen was present (CMK and CMKA) whereas pEC14pl3 prevailed under others. Correspondingly, pEC16pl1, pEC14pl2, and pEC15pl1 are IncI1 plasmids, of which pEC16pl1 was prevalent in only treatment C whereas genetically indistinguishable plasmids pEC14pl2 and pEC15pl1 were prevalent in others. This suggests that plasmids of the same incompatibility group with similar sizes and overall genetic characteristics can have drastic differences in fitness in response to the ecological condition of their hosts. In relative numbers, the IncB plasmid was statistically more abundant in comparison to other types under most conditions and the small ColRNAI-type plasmid under conditions where migration of higher-fitness hosts was absent.

Host chromosomes are known to adapt to plasmid presence, but former studies have focused on investigating the effects of singular plasmids (see, e.g., references 15, 16, and 32). Here, in an environment with 11 interacting mobilizable or conjugative plasmids (two of which were almost identical), the only consistently occurring mutations over 300 generations were observed within gene *fimE*. This gene is responsible for switching off the *fim* operon and hence fimbria production in E. coli (33). *fimE*-inactivating mutations therefore result in a continuously active fimbria operon. Fimbriae play key roles in E. coli pathogenicity, host immune responses, and biofilm production (3). IncI1 and F plasmids have been shown to encode pili that induce a fimbria-like phenotype in the host, and pilin mutants have significantly lower conjugation rates than wild type (7, 34). *fimE* inactivation and hence constitutive fimbria production are likely to be an adaptation to the general experimental conditions under which the bacteria were cultivated (similar to the study by Knöppel et al. [35]). However, many of the other adaptive mutations that Knöppel and colleagues observed were absent in our experiment or have been dominant in some other long-term experiments conducted under similar conditions (36, 37). The majority of the plasmids here encode pili with phenotypes (likely) comparable to fimbriae. Therefore, it could be speculated that the expression of chromosomally encoded fimbriae may have been under selection also due to the large number of genetically different plasmids. In other words, the production of a fimbria-like phenotype especially from the chromosome instead of one of the several potential plasmids could have had a specific benefit for the host bacterium. Perhaps those bacterial cells that produced more fimbriae than their contemporaries may have been less vulnerable to the invading plasmids that were present in the surrounding bacteria. These other bacteria may have encoded fimbria-like pili mainly from plasmids. To clarify, fimbriae or pili were present in most cells, nevertheless, and there was a difference whether it was pili that initiated conjugation or whether it was fimbriae encoded by the chromosome (which do not lead to conjugation). Along with providing a fitness advantage under culturing conditions, this chromosomal expression may have protected the bacterium from plasmid invasion and hence from the recently demonstrated acquisition-associated detrimental fitness effects (38).

Overall, the study shows that plasmids have tradeoffs that allow them to outcompete their contemporaries only under certain conditions. The big multiresistance-providing plasmids may require specific selection to remain viable in the presence of smaller competitors. This observation can be of importance, given that limiting the administration of multiple types of antibiotics reduces the selection for large plasmids like pEC14pl1 and hence may lead to their displacement by smaller plasmids with fewer resistance genes. On the other hand, broad-host-range plasmids (like RP4) may serve as reservoirs of antibiotic resistance genes in nonpathogenic hosts in different environments from which they can supply resistance to relevant hosts (as noted by Loftie-Eaton and colleagues [16]). In general, the absence of selection for plasmids via antibiotics (i.e., C, CM, and CMK) did not have an effect on plasmid prevalence. Therefore, restraining the use of antibiotics altogether does not seem to rapidly resensitize bacteria to drugs even in highly competitive situations where nonresistant, rapidly proliferating cells are present (i.e., the CMK community). Multiplasmid interactions are likely to occur in natural environments and under differing ecological settings. This work provides one of the first overviews of the dynamics that occur during such multiplasmid interactions and may illuminate situations where certain plasmids become overrepresented in relation to their contemporaries.

## MATERIALS AND METHODS

### Bacterial strains, plasmids, and culture conditions.

Bacterial strains and their plasmids are listed in Table 1. Plasmids were previously transferred from their original hosts to a second and then to a third E. coli host, in order to ensure their mobile transfer and isogenic host background (see reference 22). For initiating the community experiment, each strain was grown separately in 50-mL tubes containing 5 mL of Luria-Bertani broth (LB) (39) with the appropriate antibiotic selection (either 150 μg/mL ampicillin or 25 μg/mL kanamycin) overnight at 37°C with shaking at 200 rpm. Due to experimental design, kanamycin gene had to be inactivated in plasmid RP4. This was done as described by Ruotsalainen and colleagues (11). Briefly, a synthetic gene containing an inactivating mutation within the kanamycin resistance gene was inserted by homologous recombination in the RP4 plasmid using Red/ET recombination (Gene Bridges) according to the manufacturer’s protocol. The kanamycin-sensitive phenotype was screened from the obtained colonies, and the presence of the inactivating insertion was verified with PCR. The mutated plasmid was transferred via conjugation to HMS174.

### Plasmid competition experiment.

A microcosm experiment was set up into five ecological settings (treatments) named C, CA, CM, CMK, and CMKA. At the start of the experiment (cycle 1 [C1]), 5-μL overnight (o/n) cultures of pEC strains and RP4 were added to a 50-mL tube containing 5 mL LB medium supplemented with appropriate antibiotics and/or bacterial culture according to the setup (Table 4 and Fig. 1). The cultures were grown at 37°C and aerated by slow agitation at 60 rpm for 24 h. The experiment was done with four identical replicates/treatment. After each cycle, 50 μL of the culture (1% inoculation) was transferred to the fresh medium, and in the case of CM, CMK, and CMKA, 5 μL of overnight cultivated migrant was added. The cultures were serially propagated for 42 cycles.

**TABLE 4:** Treatments and respective conditions

Treatment	Description	Medium
C	Plasmid community without selection	5 mL LB medium
CA	Plasmid community under lethal beta-lactam selection	5 mL LB + 150 μg/mL ampicillin
CM	Plasmid community with a constant migration of plasmid-free hosts	5 mL LB + 5 μL plasmid-free HMS174 o/n culture
CMK	Plasmid community with a constant migration of higher-fitness plasmid-free hosts	5 mL LB + 2.5 μg/mL kanamycin + 5 μL HMS174(pET24) o/n culture
CMKA	Plasmid community with a constant migration of higher-fitness plasmid-free hosts under lethal beta-lactam selection	5 mL LB + 150 μg/mL ampicillin + 2.5 μg/mL kanamycin + 5 μL HMS174(pET24) o/n culture

### Sample collections and DNA extraction.

The samples were harvested after every 3 cycles in the first week and then after every 7 cycles. One milliliter of each bacterial culture (in total, 20 cultures; 5 setups × 4 biological replicates) was collected in 1.5-mL tubes and stored at −80°C for the DNA isolation. Additionally, the cultures were collected for the measuring of growth rate and maximal optical density (i.e., approximation of yield). Here, 1 mL of each bacterial culture was mixed with 300 μL of sterile 87% glycerol in cryotubes and stored at −80°C.

The total DNA (genomic and plasmid) of all the samples from cycles 1, 14, 32, 35, and 42 was extracted with the Wizard genomic DNA purification kit (Promega) following the manufacturer’s instructions. A Qubit 3.0 fluorometer was used to determine the concentration of DNA using the double-stranded DNA (dsDNA) high-sensitivity kit (Invitrogen, ThermoFisher Scientific).

### qPCR with plasmid-specific primers.

The amount of each plasmid in the samples obtained from the community experiment was quantified with quantitative PCR (qPCR). Prior to this, each primer pair used in this experiment was optimized for efficiency (see Data Set S1, sheet F, in the supplemental material). The crosscheck test with all other plasmids in this experiment was also performed to ensure the specificity (Data Set S1, sheet F). qPCR was performed in triplicates for each sample. Final reaction volume of 20 μL contained 1× SsoAdvanced universal SYBR green supermix (Bio-Rad), 0.5 μM forward and reverse primers, and 2 μL of DNA template (1 ng/μL). The qPCR cycle program consisted of initial denaturation at 98°C for 3 min, followed by 40 cycles of denaturation at 98°C for 15 s and primer annealing-extension at 60°C or 65°C (depending on the primer pairs; see Data Set S1, sheet E) for 1 min 15 s. All qPCR assays were performed in a Bio-Rad CFX96 Touch real-time PCR detection system (Bio-Rad).

The amount of each plasmid at every time point (cycles 1, 14, 32, 35, and 42) for all treatments and all replicates was analyzed with CFX Maestro 1.1 software (version 4.1.2433.1219) by normalization against 16S rRNA using normalized expression mode from gene study function.

### Bacterial growth rate and yield.

A single colony of each original bacterial strain was inoculated into 5 mL medium containing appropriate antibiotics: LB medium without antibiotics for plasmid-free HMS174, LB medium supplemented with 150 μg/mL ampicillin for HMS174 carrying pEC plasmids and RP4, and LB medium supplemented with 25 μg/mL kanamycin for HMS174 carrying pET24. Cultures were grown at 37°C, 200 rpm, overnight. Prior to the measurement of growth rate (see below), 50 μL of each culture was transferred into 5 mL of new medium and mixed thoroughly. Two hundred microliters of this inoculated medium was transferred onto a honeycomb plate with four replicates/sample. All the strains were tested for growth under four different LB-based media: without antibiotics, with 150 μg/mL ampicillin, with 2.5 μg/mL kanamycin, and with 150 μg/mL ampicillin and 2.5 μg/mL kanamycin.

Additionally, the growth rate and maximal optical density (approximation of yield) of bacterial communities at the beginning (cycle 1) and in the end of the experiment (cycle 42) from each treatment were measured. Fifty microliters of thawed sample was transferred into 5 mL of the medium composition used in the serial culture experiment (Table 4). From this, 200 μL was transferred into a honeycomb plate with four replicates/sample. The growth curves and maximum absorbance at 600 nm were measured with a Bioscreen C MBR machine (Bioscreen; Oy Growth Curves Ab Ltd.).

The maximum growth rates and approximated yields of each strain were determined with a Bioscreen C MBR machine (Bioscreen, Oy Growth Curves Ab Ltd.) at 37°C with low shaking. The absorbance was measured at an optical density at 600 nm (OD_600_) for 16 h in 5-min intervals. The maximum growth rate and yield were calculated from the data obtained from Bioscreen using RStudio (version 1.1.456).

### Conjugation rate.

All the strains with pEC plasmids and RP4 were used as a donor strain with HMS174(pET24) as the recipient. All the strains were subcultured from frozen stocks onto solid medium with appropriate antibiotic selections prior to the test. A single colony of the recipient strain and each of the donor strains was inoculated, separately, into a 50-mL Falcon tube containing 5 mL LB medium for overnight culture at 37°C, 200 rpm. The conjugation rate test, with a total of 6 different combinations, was done by mixing 5 μL of the donors and 500 μL of the recipient strain in a 1.5-mL tube containing 100 μL of LB medium. The test was done in four biological replicates for each donor. The cell mixtures were then incubated at 37°C for 2 h and shaken at 60 rpm. For the donor density checking, the cultures were plated on LB agar supplemented with 150 μg/mL ampicillin and 50 μg/mL rifampicin. The plates were incubated overnight at 37°C.

The number of transconjugants was determined by plating on LB agar with 150 μg/mL ampicillin and 25 μg/mL kanamycin and incubating the plates at 37°C overnight. The conjugation rate was calculated from CFU per milliliter of transconjugants per donor cell. Transfer of plasmids that do not encode ampicillin resistance was not studied here.

### Mutation mapping.

The DNA samples from the endpoint of the experiment were used for preparation of a sequencing library with the NEB Next Ultra DNA library prep kit (catalog no. E7370L) and sequenced on an Illumina NovaSeq 6000 platform with an S4 flow cell (PE150).

After receiving the sequence data, the mapping of plasmid sequences to find possible mutations and for comparison between treatments was performed with CLC Genomic Workbench software version 11 (Qiagen). First, the reference genomes of E. coli HMS174 and all the pEC plasmids, RP4, and pET24 were combined as a single reference file using Geneious Prime software version 2020.1.2 (Geneious). Then, the reference genomes and Illumina sequence reads of all the samples were imported into the workbench. The reads were then mapped to the references, followed by variant detection with 35% as a threshold for mutation and annotation. Finally, the data were exported to Microsoft Excel for further analysis.

### Statistical analysis.

All statistical analyses were performed using SPSS (version 26; IBM SPSS). Two-way analysis of variance (ANOVA) with Tukey HSD as a *post hoc* test was used to analyze the difference in growth rate and maximal optical density of cultures between strains and between communities under all treatments at the beginning and the end of the experiment. The effect of mobility type (MOB) and mating pair formation (MPF) on the plasmid prevalence at the endpoint of the experiment (C42), as well as the conjugation rate of each bacterial strain used in the experiment, was analyzed with one-way ANOVA, with the *post hoc* test being Tukey HSD. *P* values of <0.05 were considered significant in all tests.
